# Review of the Application of UAV Edge Computing in Fire Rescue

**DOI:** 10.3390/s25113304

**Published:** 2025-05-24

**Authors:** Hongqiang Sun, Rui Xu, Jianguo Luo, Han Cheng

**Affiliations:** 1 School of Emergency Equipment, North China Institute of Science and Technology, Langfang 065201, China; hqsun@ncist.edu.cn; 2 School of Mining Safety, North China Institute of Science and Technology, Langfang 065201, China; xu_nice_2001@163.com (R.X.); 13994357925@163.com (H.C.)

**Keywords:** unmanned aerial vehicle, edge computing, fire rescue, denied environment, cloud computing

## Abstract

The use of unmanned aerial vehicles (UAVs) attracts significant attention, especially in fire emergency rescue, where UAVs serve as indispensable tools. In fire rescue scenarios, the rapid increase in the amount of data collected and transmitted by sensors poses significant challenges to traditional methods of data storage and computing. Sensor-data processing utilizing UAV edge computing technology is emerging as a research hotspot in this field and aims to address the challenges of data preprocessing and feature analysis during fire emergency rescue. This review first analyzes fire-rescue scenarios involving UAV, including forest fires, high-rise building fires, chemical plant fires, and mine fires. Then it discusses the current status of UAV edge computing technology and its application to integrating sensor data in fire emergency rescue, analyzes the advantages and disadvantages of UAV use in fire scenarios, and identifies challenges during by UAV operations in environments with no GNSS signal. Finally, based on the analysis of fire emergency-rescue scenarios, this review argues that compared with centralized computing centers and cloud computing, distributed UAV edge computing technology based on sensor data exhibits higher mobility and timeliness and is more adaptable to the urgent nature of emergency rescue. This review also seeks to provide support and reference for the research and development of UAV edge technology.

## 1. Introduction

Fire is a widespread catastrophic phenomenon that poses serious threats globally [1]. Fires cause over 300,000 deaths annually and rank among the leading causes of accidental injuries worldwide [2]. According to China News Network, as of 13 January 2025, the Los Angeles wildfire had resulted in 24 fatalities, destroyed more than 12,000 buildings, and burned over 160 square kilometers of land. Estimates from the U.S.-based *Accurate Weather Forecasting* suggest that the multi-day fire caused economic losses estimated to be between 135 billion and 150 billion dollars [3]. The State Council of the Fire and Rescue Bureau (SCFRB) of China reported that in 2023, approximately 745,000 fires occurred nationwide, resulting in 1381 deaths, 2063 injuries, and direct property losses totaling CNY 6.15 billion [4,5].

In fire-rescue operations, the applications and contributions of aircraft have grown significantly. In recent years, the use of unmanned aerial vehicles (UAVs) in fire rescue has become particularly prominent. One advantage is that, compared to manned aircraft, a UAV flies at lower altitudes and can thus identify fire locations in low-visibility conditions, thereby reducing risks for pilots [6]. Additionally, a UAV markedly improves search-and-rescue efficiency while minimizing casualties and losses [7]. During rescue missions, a UAV is characterized by easy deployment, cost-efficient maintenance, high maneuverability, and the ability to hover in areas that may pose dangers to rescue personnel or require rapid decision-making [8]. Additionally, a UAV exhibits shorter response times to fire incidents, facilitating early-stage damage mitigation [9].

In recent years, with the rapid development of Internet of Things (IoT) technologies, computational demands in fire-rescue scenarios have grown increasingly complex and dynamic. To address these challenges, researchers proposed the concept of edge computing, which aims to enable localized data processing and analysis by deploying computational resources at the network edge. UAV edge computing technology, as a deep integration of UAV and Mobile Edge Computing (MEC), provides significant advantages in that it enhances UAV performance and ensures efficient execution of the mission.

Therefore, this paper begins with an analysis of UAV-assisted fire emergency-rescue scenarios, reviews the current state of edge computing technology and its domestic and international applications in fire-rescue operations involving UAV edge computing, and examines future challenges facing this technology. The goal is to provide foundational support and references for advancing research and development in UAV edge computing.

## 2. Scenario Analysis of UAV-Assisted Fire Emergency Rescue

With continuous technological advancements over the past decade, the use of UAVs has proliferated across various domains, such as military operations and public safety [10]. In particular, due to its high maneuverability and real-time patrol capabilities, the use of a UAV can significantly shorten response times in fire-rescue operations, thereby reducing casualties and injuries [11,12]. Based on the practical demands of fire rescue, this study analyzes four typical scenarios with distinct combustion dynamics:

- **Forest fires**: rapid propagation in open spaces.
- **High-rise building fires**: thermal-plume diffusion through vertical channels.
- **Chemical plant fires**: chain reaction with risks of deflagration of hazardous substances.
- **Mine fires:** oxygen-deficient confined-space environments.

### 2.1. Emergency Rescue Scenarios: Forest Fire

**Figure 1:**
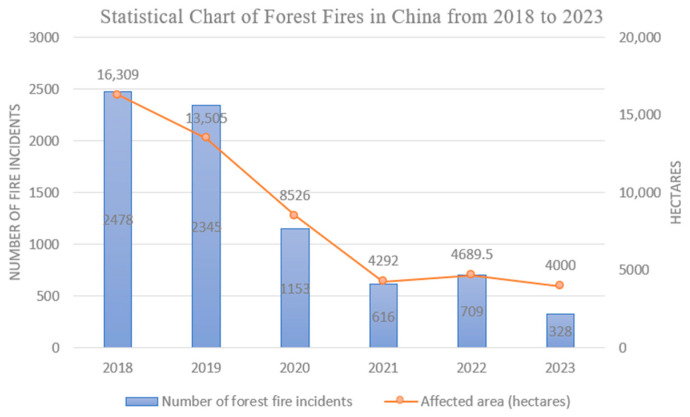
Statistical chart of forest fires in China from 2018 to 2023.

Forests, as one of the most critical natural resources, play a vital role in ecological regulation [13]. However, forest fires pose a severe threat to forest conservation. In recent years, although the frequency of forest fires in China has declined, significant losses have persisted. As illustrated in Figure 1 [14], the number of forest fire incidents dropped from 2478 in 2018 to 328 in 2023, while the affected area decreased from 16,309 hectares in 2018 to 4000 hectares in 2023. For forest fires, early detection of flames and smoke is particularly crucial [15], and the most critical factor in fire suppression is identifying fire incidents at the earliest stage [16]. UAVs, owing to their low cost, lightweight design, and high maneuverability along with other advantages, has become effective tools in forest fire emergency-rescue operations [17]. Based on an analysis of technical implementation pathways, the use of UAVs supports three core technical values in the emergency response to forest fires:

1. Fire Situation Reconnaissance and Monitoring: China’s current forest fire-monitoring system comprises three approaches: ground-based monitoring, satellite patrols, and aerial patrols. Compared to satellite remote sensing, aerial patrols offer higher ground resolution and faster response times. The rapidly advancing remote sensing technology used in UAVs introduced a new method for carrying out aerial patrols and now plays a pivotal role in forest fire prevention and control [18]. UAV-mounted cameras capture detailed real-time images of forest fires, providing updates on fire progression. Additionally, a UAV can flexibly adjust flight trajectories based on environmental conditions and mission requirements, enabling adaptive sensing and computing [19]. These capabilities significantly enhance the accuracy and timeliness of forest fire monitoring [20]. During forest fires, as flames spread and rescue personnel approach the fire zone, the fire’s morphology constantly changes. Uncertainty about the fireline’s shape may hinder decisions regarding approach routes or breakthrough points. To address this problem, ground commanders can utilize UAV reconnaissance to assist in tactical coordination. For instance, commanders can select optimal routes based on live reconnaissance footage, directing rescue teams to critical fire zones [21]. Furthermore, during fire-suppression operations, command centers can leverage the UAV to identify fire-ignition points and predict spread directions, enabling proactive risk mitigation.
2. Communication Support During Rescue: Communication is critical during forest fire incidents. However, vast forest areas often suffer from limited wireless signal coverage due to constraints on transmission distance. UAV relay systems offer an effective solution by providing temporary communication support [22]. Equipped with communication devices (e.g., radios, 4G/5G modules, satellite terminals), UAV relay systems establish temporary airborne communication networks. In multi-UAV fire rescue operations, a high-altitude UAV can act as a mobile communication base station, connecting ground devices to command centers or other nodes [22] and thereby ensuring signal coverage in otherwise unreachable areas.
3. Fire Suppression and Rescue Assistance: A UAV can carry and disperse dozens of times its weight in chemical fire retardants to create firebreaks. Under operator control, it can deploy suppression agents; for example, a UAV can launch rain-enhancement flare sticks for artificial precipitation to extinguish fires [23]. Use of a UAV also enables rapid, human-free responses to fire emergencies. Studies show that a small UAV is highly effective and safe for suppressing “cliff fires” and “flying fires” caused by “forest-block” type wildfires in harsh plateau environments. UAV-based strategies also hold reference value for suppressing fires in flat terrains [24].

### 2.2. Emergency Rescue Scenarios: High-Rise Building Fire

During urbanization, limited living space and increasing population density have led to challenges in addressing emergency fires in high-rise buildings, where chain reactions and rapid-fire spread are common. The UAV, with its excellent maneuverability, simplifies operations during search and rescue missions in high-rise fire zones and emerges as a vital force in urban firefighting. Through the lens of disaster-response paradigms, the UAV system manifests dual operational modalities for high-rise fire scenarios:

1. Precise Positioning: During rescues from high-rise building fires, the complexity and danger of the scene often prevent firefighters from entering the core fire area to obtain timely and accurate disaster information; the lack of this information poses significant challenges to suppression and rescue efforts [25]. By coordinating multiple UAVs, autonomous coverage and deployment of the fire zone can be achieved, converting non-line-of-sight (NLOS) environments into line-of-sight (LOS) conditions and thereby enabling precise target localization in fire-affected areas [26].
2. Personnel Evacuation: During high-rise building fires, dense smoke often obstructs the identification of trapped individuals. A UAV equipped with thermal-imaging cameras can detect human body-heat signatures, effectively locating trapped persons and assisting firefighters in pinpointing their positions. Simultaneously, based on real-time fire data transmitted by the UAV system, safe evacuation routes can be identified, significantly improving the rate of success in rescuing trapped individuals [27].

### 2.3. Emergency Rescue Scenarios: Chemical Plant Fire

Fire incidents are among the most common accidents in chemical plants. During chemical production, most raw materials, intermediates, and final products exhibit highly flammable, explosive, and toxic properties [28]. UAVs with vertical takeoff and landing (VTOL) and hovering capabilities [29] have demonstrated high adaptability to harsh environments and provide critical support for acquiring timely and accurate information in extreme weather or hazardous zones. To tackle the two toughest challenges in chemical plant fire emergencies, the use of a UAV provides innovative solutions, as follows:

1. Monitoring of Toxic Substances: In areas with significant toxic or hazardous material leaks, a UAV equipped with sensors for the detection of toxic substances can conduct close-range reconnaissance. By hovering over contaminated zones and performing real-time concentration measurements, the UAV can help to define containment boundaries, identify optimal entry points for mitigation of toxic material by the rescue crew, and prevent secondary accidents [30,31].
2. Fire-Scene Investigation: Chemical plant fire zones are often highly complex, with risks of structural collapses, explosions, or secondary hazards. Firefighters frequently lack comprehensive information on ignition points, explosion risks associated with storage tanks, or hazardous chemical leaks. The use of a UAV enables thorough and detailed inspections of fire scenes, as the UAV can gather critical intelligence to mitigate risks and enhance situational awareness [32,33].

### 2.4. Mine Fire Emergency Rescue Scenarios

China’s abundant coal resources play a pivotal role in the sustainable development of its national economy. However, underground fire accidents in coal mines have caused significant casualties and economic losses [34].

On 17 February 2021, a fire broke out at the Cao Jia Wa Gold Mine due to prolonged unauthorized hot work, which generated high-temperature metal slag and debris that ignited fiberglass partitions. Under the chimney effect, the fire resulted in four injuries, six fatalities, and direct economic losses of CNY 13.7586 million [35]. On 24 September 2023, a machinery failure at the Shan Jiao Shu Mine triggered a fire, leading to sixteen deaths, three injuries, and direct economic losses of CNY 42.3382 million [36].

Historically, physical and chemical methods were used to detect coal fires, but these approaches were time-consuming and hazardous. Remote sensing technology emerged to overcome these limitations, with surface-temperature detection being a representative application [37]. Airborne thermal infrared remote sensing was traditionally suitable for identifying large-scale mine fires; it struggled with smaller fires or lacked precision. In contrast, low-altitude UAV remote sensing offers high spatial resolution, frequent monitoring, and cost-effectiveness, providing a safe and rapid method for detecting thermal anomalies in dangerous or inaccessible terrains [38]. Yuan et al. [39] compared ground temperature measurements with UAV thermal-imagery data, demonstrating the high accuracy of the UAV in surface-temperature observation within coal-mining areas [40].

Compared to traditional methods, the use of a UAV can significantly reduce risks for search and rescue teams and accelerate rescue operations. However, in fire rescue scenarios such as forests, mines, high-rise buildings, and chemical plants, the use of UAVs still faces challenges and limitations. For instance, UAVs’ limited endurance remains a critical issue: UAVs rely on onboard batteries to power electronic systems and sustain flight duration, which may result in incomplete coverage of fire scenes and failure to meet large-scale rescue requirements. The introduction of edge computing technology offers a novel solution to this challenge. By shifting data-processing tasks from the cloud to the network edge, it reduces energy consumption in data transmission, thereby extending the UAV’s operational endurance.

Section 3 will delve into a comprehensive analysis of edge computing.

## 3. Advances in UAV Edge Computing Technology

In recent years, the increasing volume of data generated across various tasks necessitates processing at remote cloud data centers [41]. However, cloud computing, which relies on remote and centralized processing, faces limitations such as limited bandwidth, high latency, and high energy consumption, making it inadequate for meeting the demands of rapid-response applications in the modern era [42]. To address the bottlenecks of traditional cloud computing networks, edge computing architectures have emerged [43].

Meanwhile, advancements in computing, imaging, and networking technologies have expanded the applications of UAVs, which are evolving from single-unit operations to collaborative multi-UAV formations [6]. As the UAV is equipped with more sensors and swarm sizes grow, the volume of data collected during missions has become massive. Traditional methods face increasing constraints, which have positioned UAV edge computing as a critical focus of research in recent years.

### 3.1. Current Developments in Edge Computing Technology

#### 3.1.1. Concept and Architecture of Edge Computing

In 2013, Ryanla Mothe from the U.S. Pacific Northwest National Laboratory introduced the term “Edge Computing” in a report, marking its first formal proposal in the modern context [44]. This laid the foundation for subsequent technological evolution and widespread adoption. In May 2016, a team led by Professor Shi at Wayne State University formally defined edge computing as follows:

“Edge computing is a novel computational model that performs computing at the network edge. It processes both downstream data from cloud services and upstream data from the Internet of Everything services”.

Edge computing addresses two key challenges:

1. By processing part or all of the terminal-generated data and tasks at the edge layer rather than in the cloud, edge computing avoids prolonged latency.
2. It extracts actionable insights from massive datasets and delivers foundational services rapidly through lightweight analytics [45].

**Figure 2:**
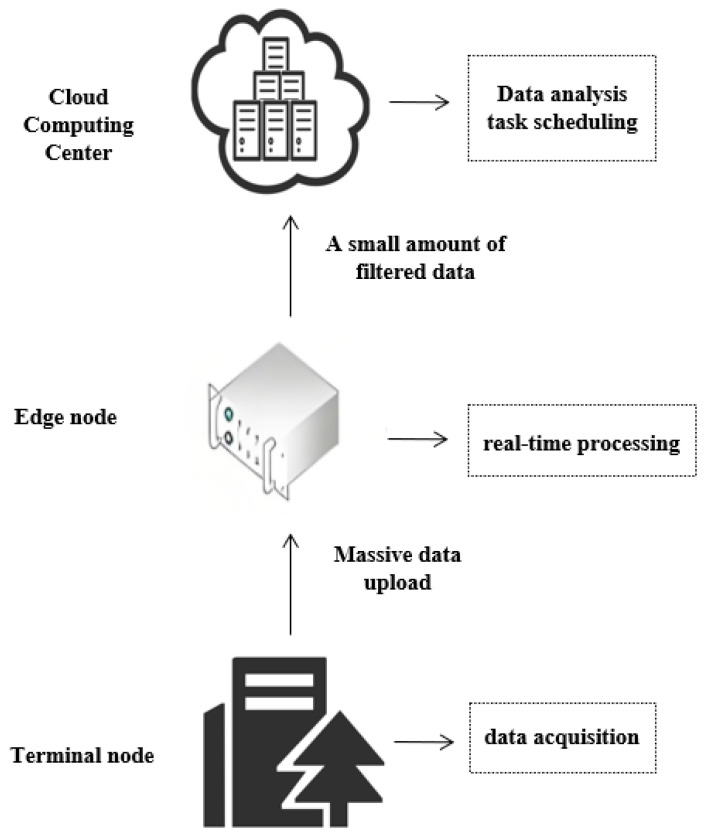
Edge computing reference architecture.

The reference architecture of edge computing, as illustrated in Figure 2, is a federated network structure that extends cloud services to the network edge by introducing edge devices between terminal devices and the cloud. This architecture is typically divided into three layers [46]:

- Device Layer: A system composed of diverse devices connected to the edge network, including sensors, actuators, fixed installations, and mobile devices. These devices are responsible for collecting a wide range of data and uploading it to the edge layer, where efficient storage and computation occur. They interface with edge-layer access points via various network types (4G, 5G, Wi-Fi), ensuring seamless connectivity between the device and edge layers and thereby enabling smooth data transmission and processing [47].
- Edge Layer: The edge layer serves as the core of the three-tier architecture and is positioned between the device layer and the cloud computing layer. Downward, it receives, processes, and forwards data from terminal devices, delivering time-sensitive services such as model training, intelligent sensing, knowledge inference, data analysis, and real-time control to users. Upward, it offloads computational workloads to the cloud for processing and retrieves results. Edge nodes often act as controllers or schedulers to manage network traffic. The edge layer comprises computing and storage devices (edge gateways, edge controllers, edge clouds, and edge sensors) and network equipment (Time-Sensitive Networking (TSN) switches and routers), encapsulating the computational, storage, and networking resources of the edge layer.
- Cloud Layer: The cloud layer receives data streams and tasks from the edge layer, processes or executes them, and returns results to the edge layer. Additionally, the cloud acts as the global controller and scheduler of the entire system, sending control directives to the edge layer to optimize network resource allocation, service deployment, and task-offloading strategies from a holistic perspective. It provides decision support and domain-specific applications such as intelligent manufacturing, networked collaboration, service extension, and personalized customization while offering interfaces for end users. Through cloud–edge collaboration, the cloud and edge layers synergize their respective strengths to enhance overall service performance [47].

With the widespread adoption of edge computing, many countries have enacted corresponding laws and standards. For example, the European Union’s General Data Protection Regulation (GDPR) mandates data minimization, user consent, data encryption, and restrictions on cross-border data transfers. China’s Data Security Law requires a classified protection system for data, obligating edge nodes to encrypt and back up critical data. Additionally, China’s GB/T 42564-2023 [48] Information security technology-Security technical requirements for edge computing specifies that edge computing developers shall ensure their systems:

(a) Support integrity verification of data during cloud–edge collaborative transmission to prevent malicious tampering during upload and download;
(b) Enable secure storage of data in cloud–edge collaboration while ensuring data integrity and availability to prevent loss or corruption;
(c) Guarantee secure distribution, processing, and destruction of cloud–edge collaborative data, permitting operations only by authorized edge computing users.

China Communications Standards Association (CCSA): Released the Industrial internet edge computing-System architecture and requirements (YD/T 4670-2024), which defines the functional and security requirements for edge nodes.

- **Functional Requirements**:
  (a) Cloud–edge collaboration;
  (b) Real-time data analysis;
  (c) Policy execution;
  (d) Alarm triggering;
  (e) Reporting of abnormal events.
- **Security Requirements:**
  (a) Security capabilities must be adapted to edge computing’s specific frameworks;
  (b) Security functions should support flexible deployment and scalability;
  (c) Security must be able to sustain resistance against attacks within a certain period;
  (d) Security must offer default support for automated implementation, with provisions for manual intervention.

#### 3.1.2. Development Status of Edge Computing Technology

The origins of edge computing can be traced back to the content delivery networks (CDNs) proposed by Akamai in 1998. CDNs improved content-distribution efficiency and reduced latency by caching content on servers near end-users, providing fast and reliable network services [44,49]. This concept laid the groundwork for relocating computational capabilities to the network edge.

In 2009, Satyanarayanan et al. [50] argued that mobile computing could seamlessly enhance human cognitive abilities through computer-intensive functions (speech recognition, natural language processing, computer vision, machine learning, augmented reality, planning, and decision-making). They introduced the Cloudlet concept—a resource-rich computing platform deployed at the network edge, connected to the internet, and accessible to mobile devices—to empower mobile users and transform diverse domains of human activity.

Before 2015, edge computing was in its early-stage technological-accumulation phase. In 2015–2017, edge computing began to gain industry-wide recognition. Beginning in 2018, edge computing entered a phase of steady and robust development.

In May 2016, the U.S. National Science Foundation (NSF) identified edge computing as a key focus area in computer systems research, replacing cloud computing in certain priorities. In August 2016, NSF and Intel held discussions on Information-Centric Networking (ICN) for wireless edge networks. By October 2016, the NSF hosted a workshop on grand challenges in edge computing, highlighting its growing importance at the U.S. government level [44].

In China, edge computing evolved in parallel with global advancements. In 2016, research institutes and internet companies established the Edge Computing Consortium. By 2018, edge computing had entered the mainstream and public awareness. In October 2018, the Cloud Native Computing Foundation (CNCF) and Eclipse Foundation collaborated to adapt Kubernetes, a technology widely used in hyperscale cloud environments, for IoT edge computing scenarios, further accelerating the adoption of edge computing [51].

From 2018–2021, edge computing found widespread applications across industries such as smart cities [52], air-traffic management [53], healthcare [54], and industrial IoT [55]. Its architecture expanded beyond traditional data centers to include intelligent edge devices such as edge computing gateways and nodes.

From 2021–2025, edge computing and cloud computing began integrating into cloud–edge collaboration models, enabling more flexible computational resource management [56]. Advances in edge technologies also made complex on-edge computation and decision-making feasible.

### 3.2. Research Status of UAV Edge Computing Technology

Edge computing technology represents a novel concept in the computing domain, bringing cloud computing services and applications closer to end users and characterized by rapid processing and shorter response times. Edge computing encompasses four models: transparent computing, Cloudlets, fog computing, and MEC [42,57]. The UAV’s flexible mobility enhances its integration with MEC services, offering greater advantages and adaptability compared to traditional MEC. This integration is particularly valuable in settings such as wilderness, deserts, and disaster zones, where ground servers struggle with rapid deployment or fail to provide timely communication and computational services. UAV edge computing effectively compensates for the limitations of conventional servers in these environments [58,59].

#### 3.2.1. UAV and MEC

Automation in UAV centers on “control”, which is fundamentally signal-based, whereas “computing” is data-driven and focuses on “strategy” and “planning”. Consequently, UAV automation primarily emphasizes scheduling, optimization, and path planning. MEC technology aligns more with this “computing” dimension. In essence, traditional automatic control relies on signal-based methods, while MEC can be conceptualized as “information-driven control” [60].

In UAV-assisted fire rescue, there is a fundamental difference between MEC and other AI algorithms, such as computer vision (CV) technology [61] and convolutional neural networks (CNN) [62], as shown in Table 1. CV and CNN technologies perform relevant computations through their corresponding algorithms, while MEC provides real-time computing-power support. The integration of MEC with other technologies can significantly enhance rescue speed in fire scenarios. Consequently, UAV edge computing technology is increasingly becoming one of the core technologies used in fire-rescue missions.

Furthermore, MEC technology enables the interconnection of multiple UAVs to form a collaborative network, facilitating autonomous flight and distributed task execution. By deploying edge computing servers in base stations that support UAV swarm operations, rapid edge-based processing of UAV-transmitted data allows real-time environmental mapping, which is then relayed back to the UAV. This closed-loop system allows the swarm to dynamically optimize route planning and obstacle avoidance [63].

#### 3.2.2. UAV Edge Computing-Related Algorithms

UAV edge computing algorithms are intelligent solutions specifically designed for UAVs using edge computing. By integrating onboard computational power with distributed edge node resources (ground base stations, neighboring UAV swarms), these algorithms enable near-source data processing, real-time task response, and optimized energy consumption. In UAV edge computing, algorithms for processing sensor data serve as core technologies for efficient perception and decision-making, requiring real-time processing, fusion, and compression of multi-source heterogeneous sensor data (visual, LiDAR, Inertial Measurement Unit (IMU), and GPS). The primary algorithm categories, with detailed descriptions of each, are given below:

- Sensor Data-Preprocessing Algorithms

Preprocessing algorithms enhance data quality and accuracy through operations like data cleaning, missing-value imputation, and outlier removal, reducing redundancy and complexity while improving analysis efficiency. These algorithms include sensor denoising/calibration and data compression/lightweight transmission [64].

1. High-frequency-noise removal using a 5×5 Gaussian kernel for image denoising;
2. Multi-scale flame color-feature extraction with dynamically adjusted clustering centers;
3. Precise flame region localization through adaptive RGB threshold segmentation.

The experimental dataset was sourced from the Bitbucket platform. This dataset, the BowFire Dataset, contains 119 annotated images. To ensure the validity of experimental results, Shen’s team [65] selected six representative images from this collection. These samples encompassed critical environmental variations, including daytime/nighttime scenarios, flame-visibility conditions (with/without obstructions), and complex background contexts. Based on this rigorously designed dataset, the proposed algorithm employs F1 score, Intersection over Union (IOU), and accuracy as evaluation metrics. The average results of ablation experiments are shown in Table 2. Experimental results demonstrated that this method achieved 97.71% accuracy (Acc) and 81.34% IOU in complex backgrounds, outperforming traditional RGB segmentation algorithms.

However, RGB-based approaches primarily depend on color models and frequently lead to false fire recognition in complex environments characterized by adverse weather conditions, inappropriate camera tilt angles, or low visibility. In recent years, with advancements in neural network technology, researchers have proposed novel fire-image-recognition methods using convolutional neural network-based image segmentation. For instance, Feng et al. [66] developed the U3U-Net model, which significantly enhances the accuracy of fire segmentation and the robustness of dynamic monitoring in complex scenarios through a nested U-shaped architecture and full-scale feature fusion.

In order to improve the quality of flame image segmentation and the accuracy of their experimental results, Feng’s team selected a custom dataset, considering images with different lighting conditions, distances, angles, backgrounds, and resolutions to enhance the performance of flame segmentation. The custom dataset contained 4535 images, split into a training and validation set. The training set contained 3908 images, while the validation set had 627 images. Experimental results demonstrated that this model achieved a processing speed of 1.5–2 fps when deployed on UAV edge devices (NVIDIA Jetson NX), meeting requirements for real-time monitoring. A comparative analysis was conducted across four dimensions: method types, core technologies, use cases, and real-time performance, as shown in Table 3.

It is noteworthy that in edge computing scenarios such as UAV-enabled fire rescue operations, the extensive deployment of IoT devices has introduced novel technical challenges. With the explosive growth of sensor-generated data, systems must operate efficiently under constrained computational capacity and storage resources while addressing latency issues caused by large-scale data transmission. This dilemma becomes particularly acute in time-sensitive contexts like fire rescue missions, where maintaining information integrity at front-end devices must be balanced with reducing communication overhead through lightweight data transmission. In this context, data compression (DC) technology emerges as a critical contributor to success. By optimizing data-representation mechanisms, DC effectively reduces data throughput while preserving informational fidelity, thereby providing a viable solution for real-time fire monitoring in edge computing environments.

The development of the DC algorithm began with Morse code, which was introduced by Samuel Morse in 1838 to compress letters in telegrams (Hamming, 1986). Morse code uses smaller sequences to represent frequently occurring letters, thus minimizing message size and transmission time. This principle of Morse code is used in the popular Huffman coding (Vitter, 1987) [67].

In UAV edge computing, data-compression technology mainly reduces the pressure on the network by reducing the amount of data to be transmitted to the cloud [68] using compression standards such as JPEG, JPEG 2000, BPG, and others [69]. Although these standards improve the efficiency of data transmission and processing, they also reduce data accuracy. The issue of JPEG and JPEG 2000 compression standards causing image distortion has been addressed. In recent years, breakthroughs in deep learning have enabled the development of intelligent compression technology that is reshaping the traditional paradigm. For example, Boehrer et al. [70] realized real-time detection and selective transmission of regions of interest (ROI) by combining hardware optimization and deep learning algorithms, applying lossless compression to key areas (e.g., moving targets) and lossy compression to backgrounds. This algorithm not only preserved the high-resolution details of key information but also significantly reduced the amount of data required to be transmitted.

- Multi-Sensor Fusion Algorithms

During the flight of a UAV, the internal systems may be affected by external disturbances such as electrical-signal interference, significant sensor drift, and strong nonlinear coupling. Additionally, process noise and observation noise are characterized by statistical uncertainty, which leads to significant sensor data errors and degradation of multi-sensor fusion performance [71]. Therefore, after sensors collect data, noise filtering and calibration are required (e.g., camera noise suppression and IMU drift compensation).

As a recursive Bayesian estimation method, the Kalman filter (KF) is one of the most commonly used core algorithms in UAV multisensor fusion navigation and positioning. On this basis, many scholars have improved it with respect to practical problems. For example, Shi et al. [72] proposed an anti-interference filtering algorithm based on an Unscented Kalman filter (UKF) to solve the stability problem caused by the interference of white Gaussian noise (WGN) on the attitude sensor data of a quadrotor UAV. Based on the linear framework of KF, the algorithm directly maps the mean and covariance of a nonlinear system through unscented transformation (UT), avoiding the error caused by linearization approximation in traditional methods. Experimental results demonstrated that under the assumption of Gaussian noise, the accuracy of the UKF for nonlinear state estimation can reach the level of a third-order Taylor expansion, which is significantly better than the second-order approximation accuracy of the extended Kalman filter (EKF). It thus effectively improves the stability of UAV attitude control in complex noise environments. Matos et al. [73] introduced the diffusion map-based Kalman filter (DMK) method to represent the uncertain noise in the environment and proposed the uncertainty- and error-aware Kalman filter (UEAKF) method to improve the accuracy of pose estimation in order to resolve the trajectory uncertainty caused by the uncertainty of noise in the UAV tracking task. The dynamic correction and noise-reduction UKF algorithm proposed by Chen et al. [74] significantly improved the accuracy of attitude estimation and robustness of UAVs in complex environments compared with traditional UKF and EKF through parameter optimization, noise coordination, and covariance correction. The aforementioned studies focus on improvements to the KF, proposing three representative optimization approaches for UAV navigation and attitude control in complex environments. Shi et al.’s anti-interference algorithm based on the UKF employs the unscented transformation to directly map nonlinear system characteristics, enhancing attitude stability under Gaussian noise. Matos et al.’s UEAKF introduces diffusion mapping to characterize noise uncertainty, improving adaptability to non-ideal noisy environments. Chen et al.’s dynamically corrected UKF achieved breakthroughs in algorithm robustness through parameter optimization and covariance correction. Although these methods all use KF as their core framework, there are significant differences in noise assumptions, nonlinear processing strategies, and optimization dimensions. Table 4 presents a comparative analysis of these three improvement methods across three dimensions: noise adaptability, algorithm complexity, and effectiveness in practical application.

By leveraging the real-time data processing capability of UAV edge computing, the process noise covariance matrix (such as the dynamic correction UKF of Chen’s team) is dynamically modified to adapt to environmental changes.

- Optimization Algorithms

Optimization algorithms such as ant colony optimization (ACO), particle swarm optimization (PSO), and A* algorithm are designed to optimize system performance by minimizing latency and energy consumption while efficiently allocating computing resources (e.g., CPU, GPU) across edge nodes. These algorithms also address real-time task scheduling, load balancing, and optimal path planning. However, each algorithm exhibits limitations. For instance, ACO requires multiple iterations to discover the optimal solution, and its computational time for large-scale problems fails to meet real-time demands in emergency rescue scenarios. PSO tends to converge to local optima, sacrificing solution diversity. A* algorithm struggles to handle dynamically changing obstacles or environmental data in real time, necessitating path re-planning for each update and compromising real-time performance.

To address these limitations, researchers have proposed improvements. For example, Hu et al. [75] investigated a UAV-assisted MEC architecture and developed an alternating optimization algorithm to jointly optimize computational-resource scheduling, bandwidth allocation, and iteration of UAV trajectory, thereby enhancing rescue efficiency. Li et al. [76] proposed a multi-UAV collaborative edge computing system. By jointly optimizing UAV fleet size, hover positioning, task-offloading strategies, and resource allocation, their approach minimized total system energy consumption. Specific methods include the following:

1. **Parameter-Adaptive Differential Evolution (PADE) Algorithm**: Dynamically adjusts parameters to optimize UAV deployment positions, maximizing coverage of IoT devices. This reduces total system energy consumption by approximately 16%.
2. **Greedy Task-Scheduling Algorithm**: Categorizes tasks and prioritizes resource-constrained tasks to ensure timely completion under latency constraints.
3. **Collaborative Optimization Framework**: Decomposes complex non-convex optimization problems into UAV deployment and task-scheduling subproblems, which are solved by PADE and the greedy algorithm, respectively.

Compared to Hu’s algorithm, Li’s approach reduces both UAV fleet size and total energy consumption through iterative optimization of UAV deployment quantity and positioning, achieving significantly lower deployment costs and lower energy usage than traditional fixed-UAV-count strategies. This advancement establishes a crucial foundation for engineering applications of UAV-enabled edge computing systems. Moreover, such cost-energy-balanced optimization methods are accelerating the transition of drone-assisted edge computing from laboratory simulations to real-world deployment scenarios.

## 4. Application of UAV Edge Computing Technology in Fire Emergency Rescue

Edge computing technology, with its low-latency response capabilities, enables rapid activation of emergency response mechanisms during fire incidents [77]. Additionally, given the UAV’s limited computing power, storage capacity, and endurance, equipping it with an MEC server can provide supplementary computational and storage resources, optimizing task-execution efficiency [78].

### 4.1. Real-Time Monitoring and Early Warning

**Figure 3:**
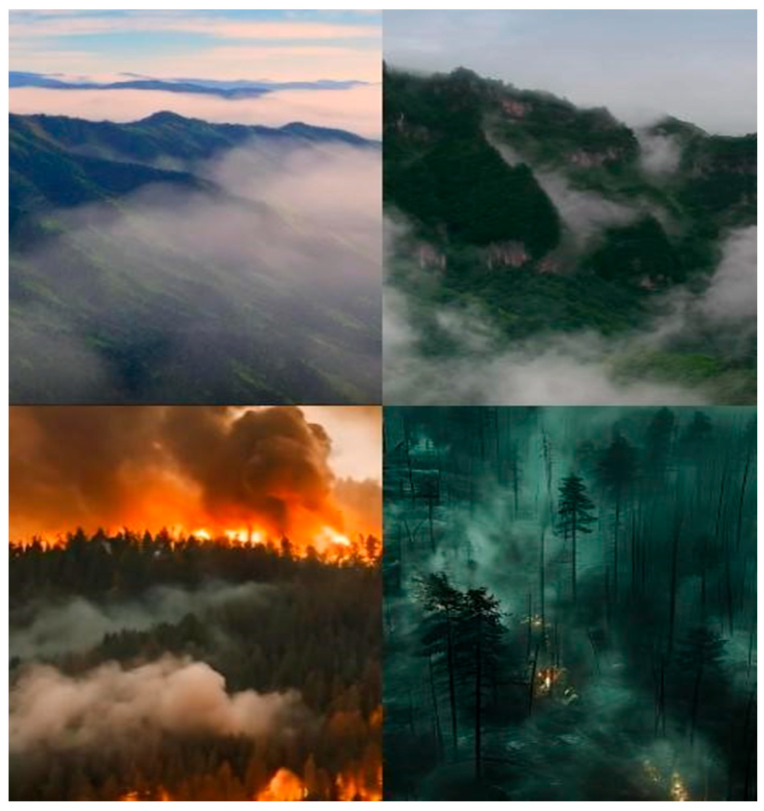
UAV takes pictures.

During real-time fire-monitoring and early-warning operations, data collected by a UAV may contain noise, redundancy, or incomplete data, as shown in Figure 3 [79]. Data-compression and -preprocessing algorithms can process the sensor data synergistically by integrating inputs from various sensors and video surveillance systems, enabling real-time monitoring of fire scenarios.

The UAV’s onboard edge computing system employs data-preprocessing algorithms to clean and denoise raw data. Subsequently, data-compression algorithms are applied to reduce the volume of processed data. Finally, the compressed data are transmitted to user servers for analysis and decision-making. For example, Zibo City in Shandong Province leveraged its core advantage of “AI+5G+computing power” to establish an air-ground integrated emergency response system, utilizing the UAV as the operational platform. Strategically deployed portable 5G signal boosters can complete temporary disaster-site network deployment within 30 min, enabling UAV transmission rates exceeding 200 Mbps. Equipping the UAV with micro edge servers allows on-device video preprocessing, reducing end-to-end latency to values as low as 8 ms. During field drills, this system demonstrated 90% accuracy in pinpointing disaster locations and proactively issuing early warnings.

The MEC server receives and analyzes the processed sensor data. When it detects fire signs such as abnormal temperature rise, smoke concentration exceeding the threshold, or flame, it sends out alarm signals; thus, it turns the ex-post evidence into a preemptive warning and passive response into active disposal, thereby improving the accuracy and timeliness of fire warning. However, in fire scenarios where temperatures can exceed 800 °C, UAVs must be equipped with high-temperature resistant materials (e.g., ceramic matrix composites) and active cooling systems, which increase their weight by 30–50% and directly reduce flight duration. In smoke-filled environments, visual sensors (RGB cameras) experience up to a 70% failure rate, necessitating the integration of infrared thermal imaging (FLIR), LiDAR (±2 cm accuracy), and millimeter-wave radar (smoke-penetration-capable radar). This need for multiple sensors increases the edge computing workload threefold to fivefold compared to baseline configurations.

### 4.2. Rescue Communication Guarantee

During natural disasters, maintaining real-time and rapid communication is crucial for improving the efficiency of rescue operations. However, in most cases, disasters such as earthquakes, tsunamis, floods, and fires often destroy the majority of ground-based communication infrastructure. For example, during Hurricane Harvey in the United States, the Federal Communications Commission (FCC) reported that only 1 in 19 base stations in Arkansas County, Texas, remained operational, with 85% of base stations in neighboring counties offline. The severe compromise of communications significantly hindered disaster-relief efforts. Therefore, establishing a rapidly responsive and adaptive emergency communication network post-disaster is critical [80].

**Figure 4:**
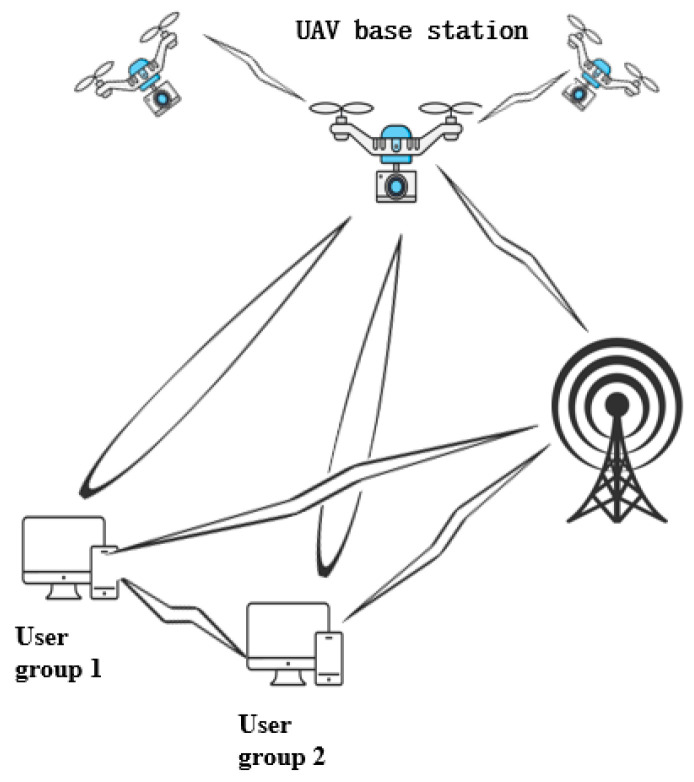
Schematic diagram of UAV base station.

In the 5G era, UAV-assisted MEC technology offers an innovative solution to this challenge. Studies by Gu [81] and Peng et al. [82] demonstrated that UAV systems equipped with MEC servers can expand computing resources for ground terminal devices through dynamic resource allocation and intelligent task-offloading mechanisms. As illustrated in Figure 4, this space–air–ground integrated architecture not only ensures low-latency communication for ground-rescue nodes but also enables flexible deployment of edge computing services. A representative case is that of the 2021 Henan catastrophic floods, where tethered UAV-mounted MEC base stations operated continuously for 72 h, successfully processing over 100,000 emergency rescue messages. This case validated the reliability and practicality of this technology in complex disaster scenarios.

### 4.3. Detection of Gas from Chemical Plants

In the event of industrial accidents or chemical contamination, the first critical information required pertains to the types and quantities of hazardous agents within the potentially affected area. Therefore, rapid reconnaissance of the target zone is essential, but the information must be acquired while minimizing human exposure risks. For example, Masuduzzaman et al. [83] addressed the limitations of aggregation node failures in wireless sensor networks and centralized data-collection techniques by proposing a UAV-based method for continuous monitoring of CO_2_ gas concentration. This approach utilizes a UAV equipped with gas sensors to continuously monitor and detect CO_2_ levels. Additionally, considering the UAV’s limited battery capacity and processing power, the method offloads sensor data from the UAV to MEC nodes for data analysis and processing tasks. When CO_2_ concentrations exceed preset thresholds, an alarm system is triggered to notify personnel to evacuate. However, in chemical plant scenarios, multiple gas types must be detected, so the UAV must carry multiple gas sensor types. To address this challenge, Maria et al. [84] proposed a UAV-mounted mobile ion mobility spectrometry (IMS) detection system designed to identify airborne toxic gases while adhering to payload and power constraints.

For example, during a national hazardous chemical rescue simulation drill, a UAV equipped with multispectral gas-sensor arrays achieved precise monitoring of high-risk toxic gases such as methanol and acrylonitrile. The edge computing system integrated models of gas-dispersion dynamics with real-time meteorological data to construct a three-dimensional spatial pollutant-dispersion-trajectory prediction system. When gas concentrations at any monitoring point exceeded preset safety thresholds, the onboard computing unit immediately activated a spatial heatmap rendering algorithm, automatically marking areas as red dynamic danger zones. Encrypted data links then synchronized tiered evacuation commands to ground command terminals and individual responder devices.

Based on the spatiotemporal dynamic risk maps provided by the system, the drill command headquarters successfully executed three tactical adjustments, ensuring operational units maintained a safety buffer distance of more than 50 m from the advancing toxic gas front. This observation effectively validated the practical value of the edge computing-enabled “monitoring-alert-decision” closed-loop response mechanism in management of hazardous chemical incidents.

## 5. Future Challenges for UAV Edge Computing Technology

UAVs have significantly improved the efficiency of rescue with its rapid response, wide coverage, real-time data transmission, and auxiliary firefighting capabilities, and it can perform tasks in complex terrain and dangerous environments, avoiding the danger of firefighters’ direct contact with the fire site and reducing labor costs and risks. However, in the fire emergency rescue scenario, a UAV may encounter limited location services [85], especially in urban high-rise areas, densely forested sheltered areas, and underground spaces. In such environments, the global navigation satellite system (GNSS) signal is typically unavailable due to signal blockage or susceptibility to interference. When the UAV’s GNSS-dependent positioning fails, target search-and-rescue operations become severely challenging [86].

In order to meet the challenge of environments with no GNSS signal, it is essential to develop and use other types of navigation systems, such as visual navigation systems [87], inertial fusion navigation systems [85], and geomagnetic fusion navigation systems [88], which pose a greater challenge to the edge computing capability of UAV.

At the scene of a fire, vision-based autonomous positioning technology enables the UAV to perceive its surroundings in real-time, achieve autonomous navigation and obstacle avoidance, and ensure safe arrival at the reach area. However, the question of how to solve the real-time calculation of UAV visual navigation and address the limits of visual navigation under dense smoke or low-light conditions [89] requires further research. The inertial navigation system relies on accelerometers and gyroscopes to measure the UAV’s motion state, making it particularly suitable for use in environments with weak or absent GNSS signals, such as high-rise-building interiors or underground spaces. Nevertheless, the UAV’s edge computing still needs to address the cumulative error caused by sensor drift over time [85] and other problems in inertial navigation fusion systems. The geomagnetic navigation fusion system positions the UAV by detecting changes in the Earth’s magnetic field, but the UAV edge computing must handle the large amount of geomagnetic data, the susceptibility of geomagnetic data to local magnetic anomalies, and the limited accuracy when used alone.

In complex communication and navigation scenarios, multi-modal-sensor-fusion navigation technology achieves centimeter-level high-precision navigation in environments with no GNSS signal through deep coupling and intelligent fusion algorithms (e.g., spatiotemporal registration and adaptive filtering) of heterogeneous sensors such as vision, inertial, and geomagnetic modules, effectively suppressing error accumulation from individual sensors. Meanwhile, intelligent reflecting surface (IRS) technology dynamically modulates the amplitude and phase characteristics of signals via passive reflecting elements, overcoming signal blockage, enhancing target signals, and suppressing interference, with microwatt-level energy consumption that reduces power usage by over a hundredfold compared to traditional relay devices. The integration of these two technologies provides a new pathway for coordinated optimization of highly reliable navigation and energy-efficient communication in UAV-enabled MEC scenarios, significantly improving system robustness and resource-utilization efficiency in complex environments.

Furthermore, there is a growing trend of edge technology performing computational tasks at the network edge. While this significantly enhances data-processing efficiency and response speed, it also introduces new threats to data security [90]. In scenarios where the UAV participates in rescue operations, the sensors it carries collect and process data that often includes highly sensitive and private information, such as fire location details, images, and video data. Such data must be transmitted to edge computing servers for processing and storage. If such data are leaked or tampered with during transmission, it could lead to severe security issues and privacy breaches.

Although traditional security technologies remain somewhat relevant, they are insufficient to comprehensively address all security threats in edge computing environments [91]. To ensure the confidentiality and integrity of data transmission between the UAV and edge computing servers, advanced encryption algorithms should be actively adopted. For instance, established technologies like the Advanced Encryption Standard (AES) and asymmetric encryption algorithms can provide robust protection for data transmission. Additionally, attention should be directed towards emerging technologies such as quantum encryption to prepare for future high-level security challenges.

For identity authentication and access control, strict authentication mechanisms are essential to secure communications between the UAV and edge computing servers. This can be achieved through digital certificates, biometric authentication, or hardware security modules. Moreover, access control policies must be tailored based on the UAV’s role, permissions, and required resources to ensure only legitimate requests are fulfilled, thereby preventing unauthorized access and data leaks.

To promptly detect anomalous or malicious activities, effective monitoring and detection mechanisms should be established. Deploying Intrusion Detection Systems (IDS) or Intrusion Prevention Systems (IPS) enables real-time monitoring of network anomalies and the implementation of defensive measures. Furthermore, leveraging machine learning and artificial intelligence technologies to analyze network traffic and system logs can enhance security monitoring capabilities, enabling more accurate identification of potential threats and attack patterns.

Ensuring that data collected and processed by the UAV during rescue operations remains secure from leaks or tampering is not only critical for respecting personal privacy and public safety but also a prerequisite for the sustainable and robust development of UAV edge computing technologies in fire rescue applications [92,93].

## 6. Conclusions

Centralized computing centers or cloud computing rely on fixed servers and data centers. Data needs to be uploaded to the remote data center for processing before results are returned. This process may be significantly delayed due to network conditions, affecting the speed and accuracy of decision-making. Because of its proximity to the data source, UAV edge computing can process the information collected by sensors in real time and conduct efficient data processing and analysis at the edge of the network, thus significantly reducing the emergency-response time. Compared to centralized computing centers or cloud computing, it reduces latency and improves bandwidth utilization.

In the fire rescue scenarios, UAV and edge computing technology can flexibly adjust tasks according to the situation on the ground and quickly adapt to the changes associated with emergencies. The application of UAV edge computing technology to fire emergency rescue has become and will remain an important trend.

## Figures and Tables

**Table 1:** The fundamental differences between AI algorithms and MEC.

Dimension	AI	MEC
Technical category	Software-level intelligent decision-making methods	Computing paradigms at the hardware–architectural level
Core objectives	Data-driven learning for prediction/classification	Complete calculation and storage near the data source
Implementation form	Mathematical model	Distributed node network

**Table 2:** Average results of ablation experiment.

	GK-RGB	RGB
Accuracy	97.71%	93.89%
IOU	81.34%	57.43%
F1-score	89.61%	72.41%

**Table 3:** Comparative analysis of GK-RGB and U3U-Net.

	GK-RGB	U3U-Net
Method type	Unsupervised Learning	Supervised deep learning
Core technology	RGB color-space threshold segmentation. Gaussian filtering denoising. Optimization of color distribution by K-means clustering.	Improved U-Net architecture. Full-scale jump connection fusion multi-level features. Residual U-shaped module.
Use cases	Flame segmentation in complex scenarios (indoor, street, etc.)	Dynamic forest fire monitoring via UAV aerial imagery
Real-time performance	Fast calculation, but weak dynamic adaptability	Support real-time monitoring and need to optimize edge deployment

**Table 4:** Comparison of Different Improved KF Algorithms.

	Improved UKF Algorithm	DMK and UEAKF	Dynamic Correction and Denoising UKF Algorithm
Noise adaptability	Improved the ability to suppress noise in complex electromagnetic environments.	The integration of the GTRS algorithm and odometer information improves the algorithm’s adaptability to noise.	In multi-sensor fusion attitude estimation, the impact of external uncertainty on the state estimation system is effectively reduced.
Algorithm complexity	The algorithm needs to run in unmanned aerial vehicle systems with high real-time requirements, so the complexity should be relatively low.	The average processing time of the algorithm is about 21 milliseconds, which translates to high real-time performance.	The algorithm increases the computational load, but it still meets the real-time requirements.
Practical application effectiveness	The algorithm has demonstrated good anti-interference performance and stability on quadcopter unmanned aerial vehicles.	Algorithms have high reliability and practicality in practical applications and can effectively guide drones to fly along predetermined paths.	The improved method significantly improves the accuracy and robustness of state estimation. Compared with the standard UKF algorithm, the average error is significantly reduced, and the algorithm can meet real-time requirements.

## Data Availability

Not applicable.

## References

[1] Zervas E., Mpimpoudis A., Anagnostopoulos C., Sekkas O., Hadjiefthymiades S. (2011). Multisensor data fusion for fire detection. Inf. Fusion.

[2] Twigg J., Christie N., Haworth J., Osuteye E., Skarlatidou A. (2017). Improved methods for fire risk assessment in low-income and informal settlements. Int. J. Environ. Res. Public Health.

[3] China News Network The Burnt Area Exceeds the Downtown Area of San Francisco! What’s Wrong with the United States When There’s No Water in the Fire Hydrant to Put out the Fire?|International Bureau of Knowledge [EB/OL]. (2025-1-13) [2025-2-06]. http://chinanews.co-m.cn/gj/2025-1-13/10352381.shtml.

[4] National Fire and Rescue Bureau The National Fire Situation Report from January to October This Year Has Been Released [EB/OL]. (2023-11-09) [2024-9-26]. https://www.119.gov.cn/qmxfxw/mtbd/spbd/2023/40340.shtml.

[5] Mukherjee A., Mondal J., Dey S. (2022). Accelerated fire detection and localization at edge. ACM Trans. Embed. Comput. Syst..

[6] Khan A., Gupta S., Gupta S.K. (2022). Emerging UAV technology for disaster detection, mitigation, response, and preparedness. J. Field Robot..

[7] Aminzadeh A., Khoshnood A.M. (2023). Multi-UAV cooperative search and coverage control in post-disaster assessment: Experimental Implementation. Intell. Serv. Robot..

[8] Lyu M., Zhao Y., Huang C., Huang H. (2023). Unmanned aerial vehicles for search and rescue: A Survey. Remote Sens..

[9] Hassler J., Andersson Granberg T., Ceccato V. (2024). Socio-spatial inequities of fire and rescue services in Sweden: An Analysis of Real and Estimated Response Times. Fire Technol..

[10] Macrina G., Pugliese L.D.P., Guerriero F., Laporte G. (2020). Drone-aided routing: A Literature Review. Transp. Res. Part C Emerg. Technol..

[11] Ouyang Q., Wu Z., Cong Y., Wang Z. (2023). Formation control of unmanned aerial vehicle swarms: A Comprehensive Review. Asian J. Control..

[12] Liu Y., Zheng C., Liu X., Tian Y., Zhang J., Cui W. (2023). Forest fire monitoring method based on UAV visual and infrared image fusion. Remote Sens..

[13] Wei X., Liu R., Liu Y. (2023). Forest change in China: A Review. Chin. Geogr. Sci..

[14] National Bureau of Statistics Annual Data [EB/OL]. (2024-02-29) [2024-10-16]. https://data.stats.gov.cn/easy-query.htm?cn=C01.

[15] Yun B., Zheng Y., Lin Z., Li T. (2024). FFYOLO: A Lightweight Forest Fire Detection Model Based on YOLOv8. Fire.

[16] Kinaneva D., Hristov G., Raychev J., Zahariev P. (2019). Application of artificial intelligence in UAV platforms for early forest fire detection. Proceedings of the 2019 27th National Conference with International Participation (TELECOM).

[17] Wang J., Fan X., Yang X., Tjahjadi T., Wang Y. (2022). Semi-supervised learning for forest fire segmentation using UAV imagery. Forests.

[18] Zhang Z., Wang B., Wu X., Zhao E. (2015). Research on fire detection methods in UAV forest fire monitoring. Remote Sens. Inf..

[19] Yu Z., Sun Z., Cheng Y., Guo B. (2024). A review of intelligent UAV swarm collaborative perception and computation. Acta Aeronaut. Et Astronaut. Sin..

[20] Wang Y., Ning W., Wang X., Zhang S., Yang D. (2023). A Novel method for analyzing infrared imagestaken by unmanned aerial vehicles for forest fire monitoring. Trait. Du Signal.

[21] Feng G. (2021). Research on the application of drones in forest fire fighting command. Saf. Prod. China.

[22] Binti Burhanuddin L.A., Liu X., Deng Y., Challita U., Zahemszky A. (2022). QoE optimization for live video streaming in UAV-to-UAV communications via deep reinforcement learning. IEEE Trans. Veh. Technol..

[23] Liang Y., Yuan X.L., Liu X.D. (2020). The application and development of drones in forest fire prevention. For. Fire Prev..

[24] Wang M., He C., Song X.F. (2022). Unmanned aerial vehicle forest fire suppression technology in high-altitude “forest block” environment. Fire Sci. Technol..

[25] Xiangs D. (2020). Research on fire fighting and rescue reconnaissance and command based on drones. China Emerg. Rescue.

[26] Li K., Huang C., Liang J., Zou Y., Xu B., Yao Y., Zhang Y., Liu D. (2023). Research on autonomous and collaborative deployment of massive mobile base stations in high-rise building fire field. Sensors.

[27] Zhoux Y. (2024). Research on the Application of drones in high rise building fire fighting. Fire Prot. Ind. (Electron. Version).

[28] Liu M., Wang Y., Luo L., Bao S., Jia B., Li X., Ding W. (2023). Segmented line heat source model for thermal radiation calculation of jet fires in chemical plants. ASME J. Heat Mass Transf..

[29] Khalili A., Rezaei A., Xu D., Dressler F., Schober R. (2024). Efficient UAV hovering, resource allocation, and trajectory design for ISAC with limited backhaul capacity. IEEE Trans. Wirel. Commun..

[30] Li P. (2022). The application of drones in chemical fire rescue. Chem. Manag.-T.

[31] Yang S.Y. (2022). application of drones in chemical fire rescue work. Chem. Manag..

[32] Wangh T. (2021). Using drones to conduct on-site fire inspections. Fire Prot. Ind. (Electron. Version).

[33] Liu C. (2022). Application of drones in chemical fire fighting and rescue work. Chem. Fiber Text. Technol..

[34] Lei K., Qiu D., Zhang S., Wang Z., Jin Y. (2023). Coal mine fire emergency rescue capability assessment and emergency disposal research. Sustainability.

[35] National Mining Safety Administration Bureau Case of the“2.17”Major Fire Accident at Caojiawa Gold Mine in Zhaoyuan City, Yantai, Shandong Province [EB/OL]. (2021-7-14) [2014-10-13]. https://www.chinamine-safet-y.gov.cn/zfxxgk/fdzdgknr/sgcc/sgalks/202107/t20210714_391956.shtml.

[36] National Mining Safety Administration Bureau Case of the “9.24” Major Fire Accident at Shanjiao Tree Mine of Guizhou Panjiang Precision Coal Co., Ltd. [EB/OL]. (2024-9-19) [2014-10-13]. https://www.chinamine-safet-y.gov.cn/zfxxgk/fdzdgknr/sgcc/sgalks/202409/t20240919_501797.shtml.

[37] Gao Y., Hao M., Wang Y., Dang L., Guo Y. (2021). Multi-scale coal fire detection based on an improved active contour model from Landsat-8 satellite and UAV images. ISPRS Int. J. Geo-Inf..

[38] He X., Yang X., Luo Z., Guan T. (2020). Application of unmanned aerial vehicle (UAV) thermal infrared remote sensing to identify coal fires in the Huojitu coal mine in Shenmu city, China. Sci. Rep..

[39] Yuan G., Wang Y., Zhao F., Wang T., Zhang L., Hao M., Yan S., Dang L., Peng B. (2021). Accuracy assessment and scale effect investigation of UAV thermography for underground coal fire surface temperature monitoring. Int. J. Appl. Earth Obs. Geoinf..

[40] Shao Z., Liang Y., Tian F., Song S., Deng R. (2022). Constructing 3-D land surface temperature model of local coal fires using UAV thermal images. IEEE Trans. Geosci. Remote Sens..

[41] Liu Z., Cao Y., Gao P., Hua X., Zhang D., Jiang T. (2022). Multi-UAV network assisted intelligent edge computing: Challenges and Opportunities. China Commun..

[42] Khan W.Z., Ahmed E., Hakak S., Yaqoob I., Ahmed A. (2019). Edge computing: A Survey. Future Gener. Comput. Syst..

[43] Carvalho G., Cabral B., Pereira V., Bernardino J. (2021). Edge computing: Current Trends, Research Challenges and Future Directions. Computing.

[44] Shi W.S., Zhang X.Z., Wang Y.F., Zhang Q.Y. (2019). Edge computing: Status Quo and Prospects. Comput. Res. Dev..

[45] Kang J., Kim S., Kim J., Sung N., Yoon Y. (2020). Dynamic offloading model for distributed collaboration in edge computing: A Use Case on Forest Fires Management. Appl. Sci..

[46] Sulieman N.A., Ricciardi Celsi L., Li W., Zomaya A., Villari M. (2022). Edge-oriented computing: A Survey on Research and Use Cases. Energies.

[47] Cheng N., He J., Yin Z., Zhou C., Wu H., Lyu F., Zhou H., Shen X. (2022). 6G service-oriented space-air-ground integrated network: A Survey. Chin. J. Aeronaut..

[48] (2023). Information Security Technology-Security Technical Requirements for Edge Computing.

[49] Pallis G., Vakali A. (2006). Insight and perspectives for content delivery networks. Commun. ACM.

[50] Satyanarayanan M., Bahl P., Caceres R., Davies N. (2009). The case for vm-based cloudlets in mobile computing. IEEE Pervasive Comput..

[51] CNCF, Eclipse Foundation Push Kubernetes to the Edge. [EB/OL]. (2018-9-26) [2024-11-22]. https://www.-sdxcentral.com/articles/news/cncf-eclipse-foundation-push-kubernetes-to-the-edge/2018/09/.

[52] Sittón-Candanedo I., Alonso R.S., García Ó., Muñoz L., Rodríguez-González S. (2019). Edge computing, iot and social computing in smart energy scenarios. Sensors.

[53] Yus S., Chenx Y. (2024). Key technological innovations and challenges in urban air mobility. Acta Aeronaut. Et Astronaut. Sin..

[54] Sacco A., Esposito F., Marchetto G., Kolar G., Schwetye K. (2020). On edge computing for remote pathology consultations and computations. IEEE J. Biomed. Health Inform..

[55] Khan F., Jan M.A., Rehman A.U., Mastorakis S., Alazab M., Watters P. (2020). A secured and intelligent communication scheme for IIoT-enabled pervasive edge computing. IEEE Trans. Ind. Inform..

[56] Xia X., Chen F., Grundy J., Abdelrazek M., Jin H., He Q. (2021). Constrained app data caching over edge server graphs in edge computing environment. IEEE Trans. Serv. Comput..

[57] Ren J., Zhang D., He S., Zhang Y., Li T. (2019). A survey on end-edge-cloud orchestrated network computing paradigms: Transparent Computing, Mobile Edge Computing, Fog Computing, and Cloudlet. ACM Comput. Surv. (CSUR).

[58] Yazid Y., Ez-Zazi I., Guerrero-González A., El Oualkadi A., Arioua M. (2021). UAV-enabled mobile edge-computing for IoT based on AI: A Comprehensive review. Drones.

[59] Li W., Guo Y., Li N., Yuan H., He M., Wang N. (2023). Intelligent reflector surface assisted UAV mobile edge computing task data maximization method. Acta Aeronaut. Et Astronaut. Sin..

[60] Songz H. (2017). Edge computing—Walking at the forefront of intelligent manufacturing (below). Autom. Expo.

[61] Bu F., Gharajeh M.S. (2019). Intelligent and vision-based fire detection systems: A Survey. Image Vis. Comput..

[62] Li P., Zhao W. (2020). Image fire detection algorithms based on convolutional neural networks. Case Stud. Therm. Eng..

[63] Zhu H., Chen Q., Zhu X., Yao W., Chen X. (2023). Edge computing powers aerial swarms in sensing, communication, and planning. Innov..

[64] Ramírez-Gallego S., Krawczyk B., García S., Woźniak M., Herrera F. (2017). A survey on data preprocessing for data stream mining: Current Status and Future Directions. Neurocomputing.

[65] Shen X., Liu Z., Xu Z. (2023). Unsupervised Flame Segmentation Method Based on GK-RGB in Complex Background. Fire.

[66] Feng H., Qiu J., Wen L., Zhang J., Yang J., Lyu Z., Liu T., Fang K. (2025). U3UNet: An accurate and reliable segmentation model for forest fire monitoring based on UAV vision. Neural Netw..

[67] Jayasankar U., Thirumal V., Ponnurangam D. (2021). A survey on data compression techniques: From the Perspective of Data Quality, Coding Schemes, Data Type and Applications. J. King Saud Univ. Comput. Inf. Sci..

[68] Azar J., Makhoul A., Barhamgi M., Couturier R. (2019). An energy efficient IoT data compression approach for edge machine learning. Future Gener. Comput. Syst..

[69] Du B., Duan Y., Zhang H., Tao X., Wu Y., Ru C. (2022). Collaborative image compression and classification with multi-task learning for visual internet of things. Chin. J. Aeronaut..

[70] Boehrer N., Gabriel A.d.S.P., Brandt A., Uijens W., Kampmeijer L., van der Stap N., Schutte K. (2020). Onboard ROI selection for aerial surveillance using a high resolution, high framerate camera. Proceedings of the Mobile Multimedia/Image Processing, Security, and Applications.

[71] Hadiwardoyo S.A., Calafate C.T., Cano J.-C., Krinkin K., Klionskiy D., Hernández-Orallo E., Manzoni P. (2020). Three dimensional UAV positioning for dynamic UAV-to-car communications. Sensors.

[72] Shi E., Xu J., Yang X., Tang C., Zhang P., Zhang D., Qin C. (2021). A new anti-interference fitering algorithm for quad rotor UAV. Proceedings of the 2021 33rd Chinese Control and Decision Conference (CCDC).

[73] Matos-Carvalho J.P., Santos R.S., Tomic S., Beko M. (2021). GTRS-based algorithm for UAV navigation in indoor environments employing range measurements and odometry. IEEE Access.

[74] Chen S., Hu F., Chen Z., Wu H. (2023). Correction method for UAV pose estimation with dynamic compensation and noise reduction using multi-sensor fusion. IEEE Trans. Consum. Electron..

[75] Hu X., Wong K.-K., Yang K., Zheng Z. (2019). UAV-assisted relaying and edge computing: Scheduling and Trajectory Optimization. IEEE Trans. Wirel. Commun..

[76] Li F., Luo J., Qiao Y., Li Y. (2023). Joint UAV deployment and task offloading scheme for multi-UAV-assisted edge computing. Drones.

[77] Shi W., Cao J., Zhang Q., Li Y., Xu L. (2016). Edge computing: Vision and Challenges. IEEE Internet Things J..

[78] Shen H., Wang D., Huang Z., Jia Y. (2024). Optimization of clustering and trajectory for minimizing age of information in unmanned aerial vehicle-assisted mobile edge computing network. Sensors.

[79] Ministry of Emergency Management of the People’s Republic of China Protecting Forests, Grasslands, Fire Prevention and Disaster Prevention Starts from Me [EB/OL]. (2024-11-21) [2025-1-10]. https://www.mem.gov.cn/kp/zrzh/202411/t20241121_512263.shtml.

[80] Wang L., Li R., Xu L., Zhu W., Zhang Y., Fei A. (2023). Aerial-Ground Cooperative Vehicular Networks for Emergency Integrated Localization and Communication. IEEE Netw..

[81] Gu X., Zhang G. (2023). A survey on UAV-assisted wireless communications: Recent advances and future trends. Comput. Commun..

[82] Peng X., Lan X., Chen Q. (2025). Age-of-Task-Aware AAV-Based Mobile Edge Computing Techniques in Emergency Rescue Applications. IEEE Internet Things J..

[83] Masuduzzaman, Nugraha R., Shin S.Y. (2022). IoT-Based CO2 Gas-Level Monitoring and Automated Decision-Making System in Smart Factory Using UAV-Assisted MEC, 2022-01-01, 2022.

[84] Allers M., Ahrens A., Hitzemann M., Bock H., Wolf T., Radunz J., Meyer F., Wilsenack F., Zimmermann S., Ficks A. (2023). Real-Time Remote Detection of Airborne Chemical Hazards—An Unmanned Aerial Vehicle (UAV) Carrying an Ion Mobility Spectrometer. IEEE Sens. J..

[85] Zhang H.J., Ma J.Y., Liu H.Y., Guo P., Deng H., Xu K. (2023). Indoor positioning technology of multi-rotor flying robot based on visual-inertial fusion. Acta Aeronaut. Et Astronaut. Sin..

[86] Wan H. (2023). UAV visual positioning method based on factor graph. Acta Aeronaut. Et Astro-Naut. Sin..

[87] Minghui L.I., Tianjiang H.U. (2021). Deep learning enabled localization for UAV autolanding. Chin. J. Aero-Naut..

[88] Gao D., Zhu M.H., Han P. (2022). A geomagnetic/inertial depth fusion navigation method. J. Chin. Inert. Technol..

[89] Zhao L., Li D., Zhao C., Jiang F. (2022). Some achievements on detection methods of autonomous landing markers for, U.A.V. Acta Aeronaut. Et Astronaut. Sin..

[90] Al-Doghman F., Moustafa N., Khalil I., Sohrabi N., Tari Z., Zomaya A.Y. (2022). AI-enabled secure microservices in edge computing: Opportunities and challenges. IEEE Trans. Serv. Comput..

[91] Kenioua L., Lejdel B., Alamri S., Ramadan Q. (2024). A password-based authentication approach for edge computing architectures. Egypt. Inform. J..

[92] Min M., Liu Z., Duan J., Zhang P., Li S. (2023). Safe-learning-based location-privacy-preserved task offloading in mobile edge computing. Electronics.

[93] Suganya B., Gopi R., Kumar A.R., Singh G. (2024). Dynamic task offloading edge-aware optimization framework for enhanced UAV operations on edge computing platform. Sci. Rep..

